# Arctic warming interrupts the Transpolar Drift and affects long-range transport of sea ice and ice-rafted matter

**DOI:** 10.1038/s41598-019-41456-y

**Published:** 2019-04-02

**Authors:** Thomas Krumpen, H. Jakob Belter, Antje Boetius, Ellen Damm, Christian Haas, Stefan Hendricks, Marcel Nicolaus, Eva-Maria Nöthig, Stephan Paul, Ilka Peeken, Robert Ricker, Rüdiger Stein

**Affiliations:** 1 Alfred Wegener Institute, Helmholtz Centre for Polar and Marine Research, Am Handelshafen 12, 27570 Bremerhaven, Germany; 2 Ludwig Maximilians University, Department of Geography, Luisenstraße 37, 80333 Munich, Germany

## Abstract

Sea ice is an important transport vehicle for gaseous, dissolved and particulate matter in the Arctic Ocean. Due to the recently observed acceleration in sea ice drift, it has been assumed that more matter is advected by the Transpolar Drift from shallow shelf waters to the central Arctic Ocean and beyond. However, this study provides first evidence that intensified melt in the marginal zones of the Arctic Ocean interrupts the transarctic conveyor belt and has led to a reduction of the survival rates of sea ice exported from the shallow Siberian shelves (−15% per decade). As a consequence, less and less ice formed in shallow water areas (<30 m) has reached Fram Strait (−17% per decade), and more ice and ice-rafted material is released in the northern Laptev Sea and central Arctic Ocean. Decreasing survival rates of first-year ice are visible all along the Russian shelves, but significant only in the Kara Sea, East Siberian Sea and western Laptev Sea. Identified changes affect biogeochemical fluxes and ecological processes in the central Arctic: A reduced long-range transport of sea ice alters transport and redistribution of climate relevant gases, and increases accumulation of sediments and contaminates in the central Arctic Ocean, with consequences for primary production, and the biodiversity of the Arctic Ocean.

## Introduction

**Figure 1:**
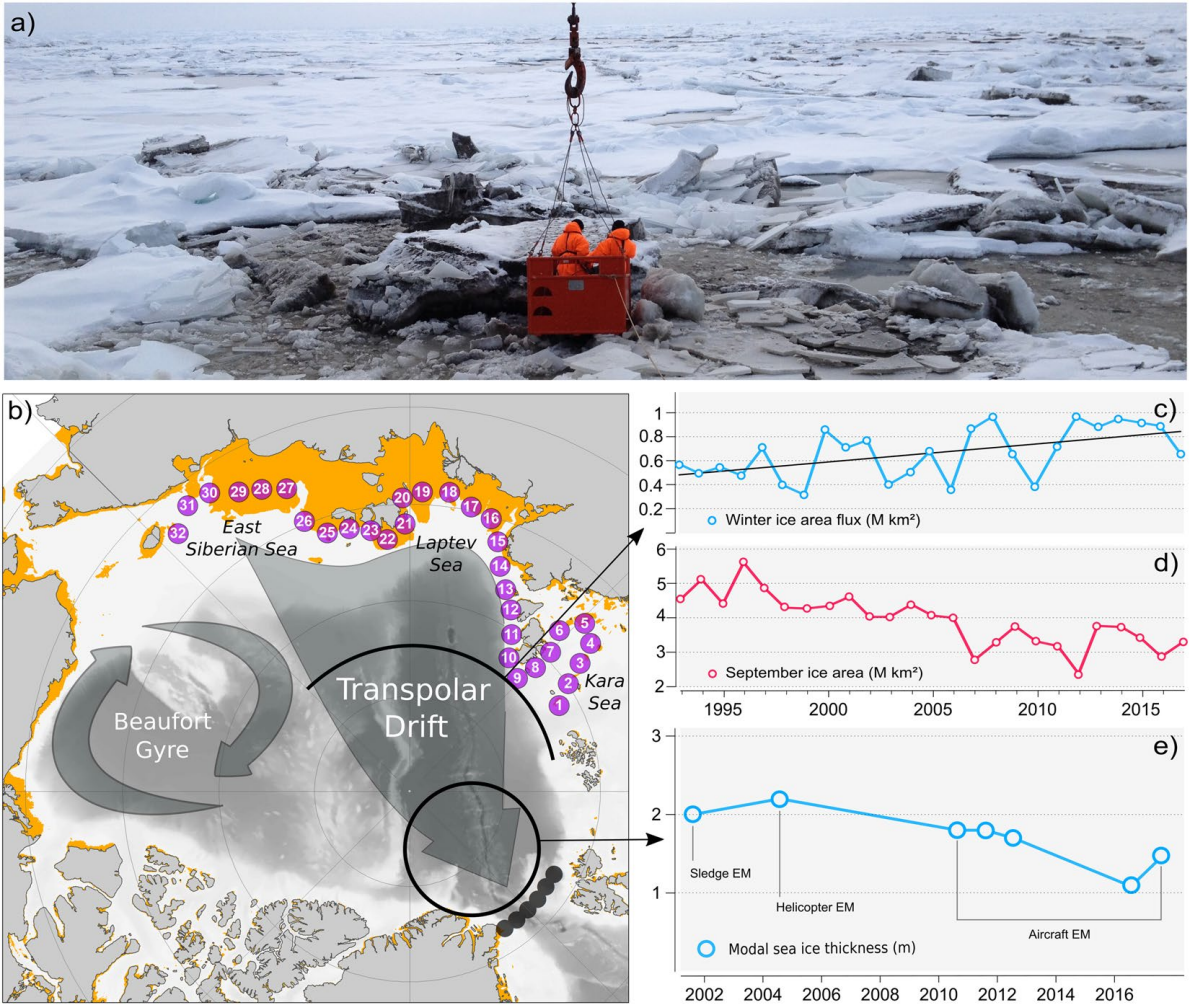
(**a**) Photo of ice-rafted material (sediments) observed during a RV *Polarstern* cruise in the Transpolar Drift (PS87 at 86.68°N, 148.75°E in 2014). (**b**) Overview map of the Arctic Ocean. Orange zones along Arctic coastline indicate shallow water areas of less than 30 m water depth where entrainment of biogeochemical material takes place. Subsequently, ice-rafted material is transported by the two major drift regimes: The Beaufort Gyre, a clockwise circulation regime and the Transpolar Drift, transporting sea ice from the Siberian Shelf Seas towards Fram Strait. To determine drift patterns and source areas of sea ice that exits the Arctic between Greenland and Svalbard, backward trajectories were calculated starting from six positions located in northern Fram Strait (dark grey circles). In a second experiment, we investigated the survival rates of sea ice that was formed in the Siberian Shelf Seas by forward-tracking sea ice starting from the 32 purple circles located along the coast of Siberia. Purple circles mark shallow regions with water depths between 25–30 m. (**c**) Time series of winter (October–April) ice area flux across a zonal gate positioned along 82.5°N, 60°E–180°E (black line). (**d**) Time series of summer (September) sea ice area (source NSIDC), (**e**) time series of sea ice thickness North of Fram Strait from Electromagnetic (EM) sounding between 2001–2018 (source AWI *IceBird* program).

Arctic sea ice acts as a transport medium for climate relevant gases, macro-nutrients, iron, organic matter, sediments, and pollutants^1–5^ (Fig. 1a). In addition it serves as a habitat for sea ice biota, which are an important food source for the pelagic ecosystem^6^. A large fraction of the ice-rafted matter as well as organisms are incorporated into newly formed sea ice on the wide Arctic shelves with average water depths of less than 30 m^7,8^. Entrainment is primarily controlled by suspension freezing in frazil ice during freeze-up, in polynyas^9,10^ and, to a smaller degree, by grounded sea ice pressure ridges plowing through the sea floor^11^. After formation, offshore-directed winds push the ice away from shallow coastal zones towards the central Arctic Ocean. First-year ice (FYI) that survives summer melt and turns into second-year ice (SYI) is transported further and contributes to the long-range transport of biota and biogeochemical material across the Arctic. Under modern (interglacial) conditions, in particular the Transpolar Drift (Fig. 1b) plays a crucial role for the Arctic sedimentary budget and biogeochemical cycles^12^. It moves ice-rafted material with an average speed of 7 km per day from the Siberian shelves over the Central Basin towards Fram Strait where the ice leaves the Arctic Ocean and melts.

Predicted future changes towards a seasonally ice-free ocean^13,14^ will, at some point, cut-off long-range transport of ice-rafted materials by the Transpolar Drift. Studies examining long-range transport of sea ice over the past decades are scarce, and the few existing investigations rather indicate an increase in exchange of ice-rafted material between regions. The increase in exchange is associated with a faster sea ice drift^15^ resulting from a thinning ice cover, which breaks up more easily by wave and wind forces. Consequently, more ice is formed in polynyas along the coast^16^ and hence, more ice^17^ and potentially more ice-rafted material is advected from the Siberian Shelf Seas to the central Arctic Ocean and Fram Strait (Fig. 1c).^18^ expect that continued fall and winter ice production coupled with higher ice velocities are likely to ensure continuity of the long-range transport.

However, during the past few years, summer ice extent in the marginal ice zones was particularly low, so that most ice exported from the shelves had melted before it could be included in the Transpolar Drift (Fig. 1d). In this study we investigate if intensified summer melt in marginal ice zones is strong enough to cut-off transport pathways much earlier than expected, despite faster drift velocities. We assess interannual variability and trends in long-range transport using state-of-the-art, satellite-derived ice drift products. Furthermore, we identify potential sites of melt out and intensified release of ice-rafted substances and discuss consequences for the sedimentary budgets, biogeochemical cycle and the ecosystem.

## Results

### From the Siberian shelves to Fram Strait

**Figure 2:**
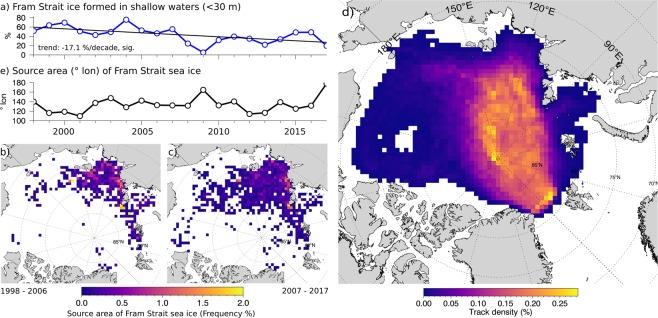
Results from backward-tracking of sea ice starting from 6 locations in Fram Strait (shown in Fig. 1b) between 1998–2017. Tracking was initiated at a two-week interval. The ice is tracked backward in time until it reaches land or fast ice or until sea ice concentration drops below 20%. (**a**) shows the fraction (averaged annual frequency) of sea ice leaving Fram Strait that originates from shallow shelf areas with less than 30 m water depth. The two maps (**b**,**c**) show the formation sites of sea ice leaving Fram Strait gridded on a 62.5 × 62.5 km grid: (**b**) for the period between 1998–2006 and (**c**) between 2007–2017. Ice younger than 2 month was excluded from the analysis. In (**d**) the gridded density of all backward trajectories is shown. The typical course of the Transpolar Drift is emphasized by high track frequencies. (**e**) Provides the annual averaged origin (° longitude) of Fram Strait sea ice.

To examine interannual variability and trends in sea ice pathways and survival rates, we applied a Lagrangian sea ice tracking approach. The same approach was used in various studies before^4,5,19–21^ and is capable of tracing sea ice backward or forward in time based on multiple satellite-derived sea ice motion products (see Material and Methods). The analysis of 20 years (1998–2017) of backward trajectories starting in Fram Strait reveals that less and less ice formed in shallow water areas (<30 m) has reached Fram Strait (−17% per decade, Fig. 2a). The trend is significant at the 95% confidence level. Instead, the proportion of Fram Strait sea ice formed during freeze-up in deeper waters of the Arctic Ocean has increased (compare Fig. 2b,c). Ice that has formed outside the well-mixed shallow waters carries less ice-rafted materials. This suggests a reduced long-range transport of biological and biogeochemical matter towards the central Arctic Ocean and Fram Strait, despite accelerating drift speeds and increasing ice-area fluxes.

The main source region for ice-rafted material in the Transpolar Drift and Fram Strait is the Laptev Sea (Fig. 2d). The majority of sea ice originates from coastal areas between 110°E and 150°E with only small interannual variabilities (Figs 2e and S3a). Only during years characterized by unusual drift patterns, Fram Strait outflow is temporarily dominated by sea ice coming from the East Siberian Sea and the Beaufort Gyre (2009 and 2017). Our findings are consistent with other satellite-based studies of sea ice trajectories^22^, and analyses of mineralogical characteristics and source areas of surface sediments from the central Arctic Ocean and Fram Strait area^2^.

The overall lack of a trend and the low variability in Fig. 2e indicates that the reduced long-range transport cannot be explained by a shift in large-scale patterns of ice dynamics. Evidence that the decreasing long-range transport is rather related to an intensification of sea ice melt, is provided by sea ice thickness surveys conducted North of Fram Strait within the framework of the AWI *IceBird* program (Fig. 1e): The thickness of undeformed level sea ice (modal thickness) reaching Fram Strait has steadily decreased since 2001^20,23^, while the age of surveyed ice does not differ much for the surveying years. Sea ice covered by EM surveys between 2001 and 2018 originates primarily from the Laptev Sea and reached Fram Strait 2–3 years after it was formed. Responsible for the observed thinning are rising atmosphere and ocean temperatures in the source areas of sea ice, later onset of freeze-up, earlier onset of melt^24^, and increased ocean mixing^25^. The observed increase in sea ice drift velocities (Fig. S3b) and a reduction of sea ice age in Fram Strait (Fig. S3c) further reduces time for sea ice to grow.

### Survival rates and melt sites of sea ice from Russian shelves

**Figure 3:**
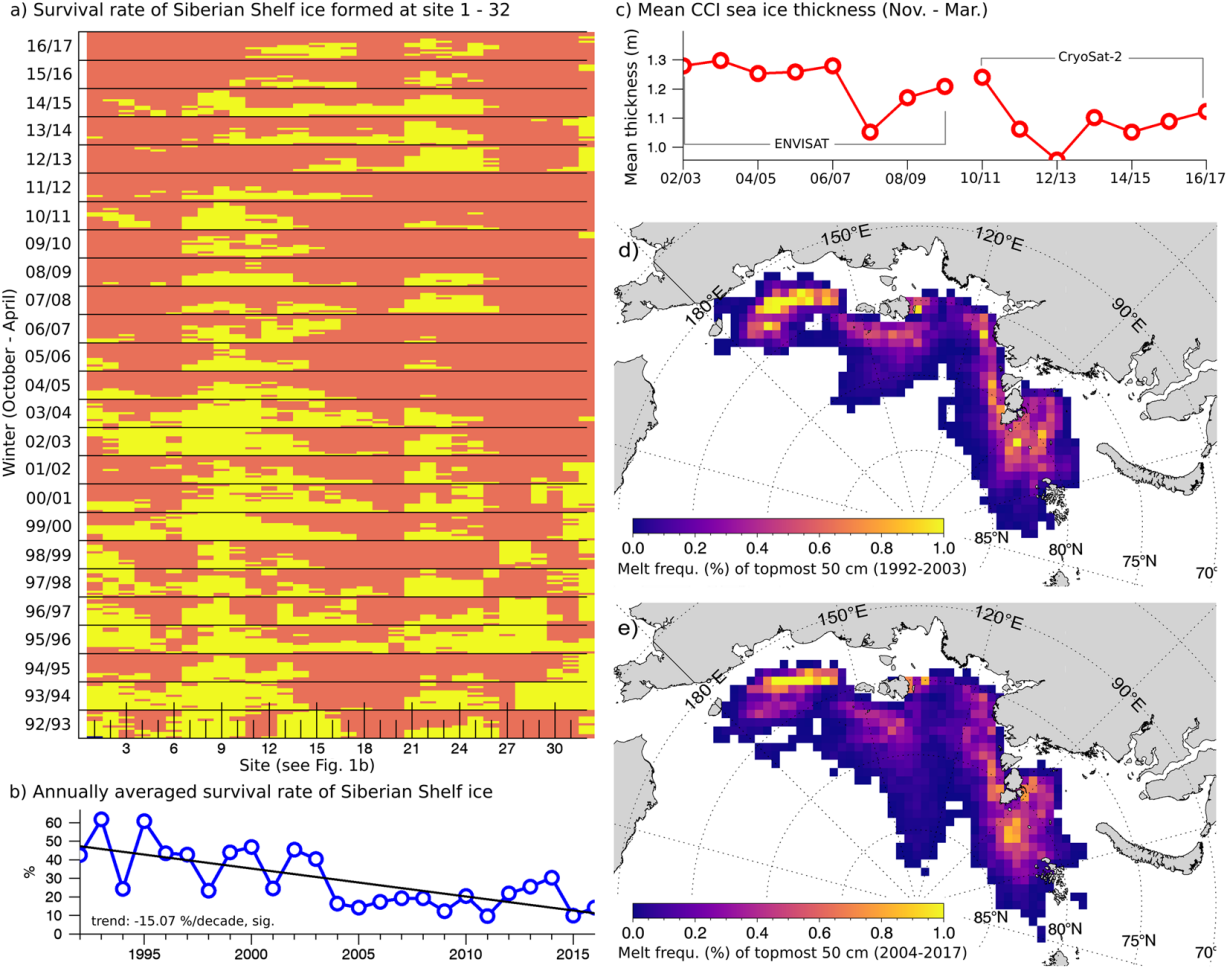
Utilizing the forward tracking we examined the amount of sea ice, originating from the Siberian shelf (<30 m), that survived the first summer. (**a**) Shows whether ice formed in winter (y-axis, October–April) between 1992 and 2017 at one of the 32 points (x-axis) melts in summer (red), or survives until next freeze-up (yellow). Ice that survives summer is incorporated into the Transpolar Drift and contributes to long-range transport. The mean annual survival rate averaged over all points is shown in (**b**). The annually averaged 120 day mean thickness (November – March) of sea ice formed in November between site 1–32 (compare Fig. 1b) is shown in (**c**). Along-track mean sea ice thickness estimates were obtained from CryoSat-2 and ENVISAT missions. The two maps show where the remaining topmost 50 cm of FYI melts during summer month: (**d**) for the period between 1992–2003 and (**e**) between 2004–2017.

To assess the melt probability of first-year ice (FYI) and to identify regions of most pronounced melt, a second experiment was carried out. Again, we used the Lagrangian approach to trace ice forward in time from 32 points located along the Siberian coast at a water depth favorable for suspension freezing (Fig. 1b, 25–30 m). The tracking stopped when FYI completely melted or turned into second year ice (SYI, see Material and Methods). Figure 3a,b show the significant decrease in the survival probability of ice that formed along the shallow coastline (−15% per decade). While in the 1990s up to 50% of FYI entered the Transpolar Drift, rarely more than 20% survived summer melt 15 years later to drift across the Arctic Ocean. Whether newly formed ice contributes to long-range transport or melts during summer months depends upon the geographical location and time of formation, and the annual weather patterns. Sea ice that formed in fall over shallow areas located closer to the pole (e.g. Komsomolez Islands) is more likely to survive the summer melt than ice formed in late winter in the deep embayment of the Laptev Sea (Fig. 3a and Table S1). A decrease in the survival probability of first-year ice is recognizable at all points, but significant only in the Kara Sea, East Siberian Sea and western Laptev Sea (Table S1).

If the reduced probability of FYI survival is accompanied by a thinning ice cover is difficult to assess, because direct airborne observations are only available for Fram Strait, but not for the Russian Shelves. However, the satellite-based Sea Ice Thickness Climate Data Record (SIT CDR v2.0, see Material and Methods) from the ESA Climate Change Initiative (CCI) indicates a decreasing winter mean ice thickness in the past 15 years (Fig. 3c). Following^26^, the thinning FYI cover is the consequence of enhanced offshore-directed transport (compare Fig. 1c), because during winters dominated by an offshore-directed drift component, newly formed ice areas stay rather thin and may melt more rapidly once temperatures rise above freezing. In contrast, new ice zones formed during winters with enhanced onshore advection are subject to a stronger dynamic thickening, which in turn delays the onset of sea ice retreat. The thinning winter ice cover, together with enhanced offshore-directed export, preconditions early ice retreat and low summer ice extent. Both factors favor the decline of long-range transport of ice-rafted material.

The evolution of sea ice, namely, growth from the underside in winter, and sea ice melt at the top in summer causes ice-rafted particles to migrate towards the ice floe surface^27^. To identify potential sites of melt out and intensified release of biogeochemical matter, we used a simple thermodynamic model. The model calculated level ice growth and melt along trajectories based on surface temperatures, ocean heat flux assumptions and a snow climatology (see Material and Methods). Figure 3d,e show where during summer month, the remaining topmost 50 cm of ice formed along the Siberian coast melted. The period from 1992–2003 (Fig. 3d) was characterized by higher survival rates of FYI, while the 2004–2017 period (Fig. 3e) showed reduced survival probabilities. Assuming that large quantities of biogeochemical matter accumulate near the surface (e.g. sediments, pollutants, iron), the past decade was characterized by intensified sedimentation/release of biogeochemical matter in the central Arctic Ocean and north of the Laptev Sea.

## Discussion

Because^28^ of their arctic-wide spatial coverage with a constant temporal resolution, low-resolution sea ice motion products from satellites have been widely used in sea ice studies and for model assimilation^15,28–30^. It stands to reason that validity and reliability of the presented results from trajectory studies depend on the accuracy of the applied sea ice motion product. Therefore, tracking experiments were repeated using different combinations of motion products, as well as higher ice concentration thresholds (e.g. 40%), but a reduced long-range transport was observed independent of the forcing dataset and the chosen setup.

In this study, we primarily use the CERSAT drift dataset to calculate trajectories, because it provides the most consistent time series of motion vectors starting from 1991 to present. Comparisons with buoys and high resolution SAR images indicate that in particular during winter months, when the atmospheric moisture content is low and surface melt processes are absent, the quality of motion products from low resolution satellites is high^31–33^. Restrictions may arise from the coarse resolution of the sensors in near-shore regions characterized by a complex coastline, extensive fast ice areas, and polynyas^31^. However, a cross-comparsion with drift estimates obtained from long-term moorings deployed on the Laptev Sea shelf shows that CERSAT motion products are capable of reproducing sea ice drift in near-shore areas correctly^17^.

During summer months, strong surface melt processes and high moisture content in the atmosphere further reduces accuracy of motion vectors and lead to an underestimation of the ice drift^28^. This effect is particularly pronounced at the ice edge and in Fram Strait. Because CERSAT drift estimates are not available between June and August, motion information is bridged with OSISAF or NSIDC data (see Material and Methods). At this point, it should be noted that the lack of temporally consistent climate data records of sea ice drift covering all seasons makes it difficult to carry out, validate and compare studies such as this one. To quantify uncertainties of sea ice trajectories, we therefore reconstructed the pathways of 57 drifting buoys obtained from the SeaIcePortal.de (Fig. S1)^34^. Overall we find that the deviation between actual and virtual tracks is rather small (36 ± 20 km after 200 days) and considered to be in an acceptable range. Moreover, Fig. S1 does not show any indication that with the onset of surface melt in summer the error increases significantly (red lines). Only if buoys enter Fram Strait (dashed lines), distance between real and virtual buoys is gradually increasing. Note that the trajectory error after two or three years of ice drift cannot be properly assessed because buoys hardly survive more than one year due to summer ice melt and instrument failures.

### Impact on sedimentary budgets, biogeochemical cycle and ecosystem

By combining Lagrangian trajectories with other state-of-the-art satellite and model products, we established a new tool to investigate changing Arctic sea ice and processes acting on the ice cover along pathways. In this study we show that long-range transport from shelf seas towards Fram Strait declines. Instead, there is evidence of intensified melt in the central Arctic Ocean and north of the Laptev Sea. To assess related changes in sedimentation rates of ice-rafted materials, reliable estimates of material uptake in shallow waters are needed. At present, estimates are still scarce – particularly because incorporation of ice-rafted materials into sea ice during formation or at a later stage is a very complex process that depends on the entrainment process itself, water masses, depth and stratification, and the presence of emitting sources (river outflow, thawing permafrost, etc.) in the surrounding waters^35^. However, consequences for the sedimentary budget, biogeochemical and ecological effects can be assessed for some materials that are studied more regularly.

For example ice-rafted sediments in Arctic sea ice have received much attention since they may reflect recent and past climatic and environmental conditions of the Arctic Ocean and its marginal seas and serve as a proxy to identify source areas of sea ice^36,37^. Following^3,8,12^, the annual sea ice sediment uptake in the Kara Sea, Laptev Sea and East Siberian Sea is around ~34 × 10^6^ t y^−1^ (see Fig. S4). By means of the FYI survival rate from the forward tracking approach we can make a simple quantification of the sedimentation rates before and after 2003. The sedimentary budged (Material and Methods) given in Fig. S4, and Table S2 provides evidence that after 2003, 24% (~4.8 × 10^6^ t y^−1^) more sediments are released in the marginal ice zones and central Arctic Ocean due to intensified melt of FYI. The largest part is released close to where the ice was formed (compare Fig. 3d) but there is evidence of an intensified sedimentation/melt in the central Arctic Ocean and north of the Laptev Sea (Fig. 3e). Our quantitative estimates support recent findings of ^38^, showing that Radium-228, a tracer for sediment-water exchange processes, has significantly increased in the central Arctic over the past decade. The authors assume that this increase is due to an intensification of shelf-derived material inputs to the central basin by surface waters and sea ice. Moreover, the trend of declining long-range mineral transport from the shelves to Fram Strait is supported by observations from sediment traps. Between 2000 and 2009 biomarkers for terrigenous origin show a continuously decrease in Fram Strait, while between 2000 and 2012 biogenic matter increased^39^.

The observed significant changes in transport range of the Transpolar Drift may also impact primary production in Arctic sea ice. Because light availability under sediment-laden sea ice is significantly reduced, development of bottom ice algal blooms is delayed or inhibited compared to clean ice conditions^40^. Hence, a decreasing fraction of sediment-laden ice downstream in the Transpolar Drift, may contribute - along with the general thinning of ice (Fig. 3c) to enhanced light availability and primary production^39,41,42^. While primary production increases, the biodiversity of sea ice biota in the central Arctic Ocean may be negatively affected by the rapid melt and decreasing long-range transport. Less seeding cells would be transported from shallow shelves, and the loss of the more diverse SYI may significantly change bacterial and sea ice algae assemblage^43,44^.

Impacts can also be expected for the sea ice-ocean-atmosphere coupling. Sea ice transports methane taken up especially from polynya regions on Siberian shelves^4^. Our results reveal that only ice formed in polynyas between October and January is advected far enough North to survive summer and being incorporated into the Transpolar Drift. Ice that is formed later is melting during summer month. Therefore a disconnected shelf-ocean sea ice transport also fails to transport methane away from the shelf to the interior Arctic. As a result the shelf water will be charged with methane surplus during the ice-free summer season, whereas today the methane surplus is low in the Laptev Sea^45^.

In summary the observed reduction of long-range transport due to the accelerated sea ice retreat are expected to change biogeochemical fluxes and ecological processes in the Central Arctic. Pollutants such as microplastics and other contaminant may accumulate in the central Arctic or are released in the marginal ice zones, where they concentrate in the higher trophic levels of the food web^46^. Quantitative assessments along the Transpolar Drift from the shelf seas to Fram Strait are needed to further investigate the biogeochemical consequences of the declining long-range transport.

## Material and Methods

### Sea ice trajectories

To determine sea ice drift trajectories we developed a Lagrangian approach (ICETrack) that traces sea ice backward or forward in time using a combination of satellite-derived low resolution drift products. So far, ICETrack has been used in a number of publications to examine sea ice sources, pathways, thickness changes and atmospheric processes acting on the ice cover^4,5,19,20,47^.

Sea ice motion information is provided by different institutions, obtained from different sensors, and for different time intervals^28,15,28–30^. In this study we apply a combination of three different products: (i) motion estimates based on a combination of scatterometer and radiometer data provided by the Center for Satellite Exploitation and Research (CERSAT)^32^, (ii) the OSI-405-c motion product from the Ocean and Sea Ice Satellite Application Facility (OSISAF)^33^, and (iii) Polar Pathfinder Daily Motion Vectors from the National Snow and Ice Data Center (NSIDC)^48^.

The tracking approach works as follows: An ice parcel is traced backward or forward in time on a daily basis. Tracking is stopped if a) ice hits the coastline or fast ice edge, or b) ice concentration at a specific location drops below 20% and we assume the ice to be melted (forward tracking) or formed (backward tracking). A weighted approach is used to determine the motion product that is applied for the tracking: The algorithm first checks for the availability of CERSAT motion data within a predefined search range. CERSAT provides the most consistent time series of motion vectors starting from 1991 to present and has shown good performance on the Siberian shelves^17,31^. During summer months (June–August) when drift estimates from CERSAT are missing, motion information is bridged with OSISAF (2012 to present). Prior 2012, or if no valid OSISAF motion vector is available within the search range, NSIDC data is applied. For a detailed description of the differences between the CERSAT, OSISAF and NSIDC products we refer to^28^.

### Experimental setup for backward tracking of sea ice leaving Fram Strait

In order to determine drift patterns and source areas of sea ice that exits the Arctic between Greenland and Svalbard, backward trajectories were calculated starting from six positions located in northern Fram Strait (81.3°N, 10°W–15°E, shown in Fig. 1b, dark grey circles). Trajectories are computed every two weeks between 1998 and 2017 (January–December) with a maximum duration of seven years. Backward trajectories of all sea ice exported through Fram Strait during the 1998–2017 period are shown in Fig. S2a).

### Experimental setup for forward-tracking of sea ice formed on the Siberian shelf

The survival rate of sea ice formed on the Siberian shelf sea is investigated by tracking sea ice in a forward direction starting from the 32 points shown in Fig. 1b). The 32 points are located along the Siberian coast offshore the fast ice edge where waters are between 25–30 m deep. Sea ice was tracked at two-week intervals from October–April between 1992 and 2017 until next freeze-up. Forward trajectories of all sea ice formed on Siberian Shelf seas during the 1992–2017 period are shown in Fig. S2b).

### Water depth, sea ice concentration and thickness along trajectories

Water depth along trajectories is extracted from the International Bathymetry Chart of the Arctic Ocean (IBCAO)^49^. The applied sea ice concentration product is provided by CERSAT and is based on 85 GHz SSM/I brightness temperatures, using the ARTIST Sea Ice (ASI) algorithm. The product is available on a 12.5 km × 12.5 km grid^50^. Sea ice thickness information of the winter month between October and April (Fig. 3c) is based on the sea ice thickness climate data record from the ESA Climate Change Initiative (CCI). The gridded thickness fields are derived from freeboard trajectory data from the radar altimeters onboard the Envisat and CryoSat-2 satellite platforms in the period from 2002 to 2017. The climate data record provides the mean sea ice thicknesses of the ice-covered area in each grid cell with a spatial resolution of 25 km and a duration of 1 month. The spatial coverage of Envisat sea ice thickness observation is limited to latitudes below 81.45°N due to the orbit parameters of the satellite, while the orbit of CryoSat-2 allows observations up to a latitude of 88°N. The data sets and algorithm documentation are available at the CCI Data Portal^51,52^.

### Thermodynamic ice growth along trajectories

Along trajectories, a simple one-dimensional thermodynamic model calculates sea ice growth and melt. The model was developed by^53^ and has been used by^54^ to reconstruct the surface hydrography of the Arctic Ocean interior. Here we use the model output to number accumulated melt rates and identify potential sites where intensified sedimentation on Siberian Shelf Seas and in the central Arctic Ocean takes place (Fig. 3d,e). The model computes thickness changes at daily increments based on surface air temperature, ocean heat flux and snow cover. Surface air temperature, together with surface level pressure and 10 m wind, are extracted along the ice trajectories from NCEP re-analysis^55^. Snow depth is computed from the Warren climatology^56^, while the ocean heat flux is assumed to be constant at 2 W/m². The model was validated against ice-mass-balance buoys^57^ providing an accuracy of a few centimetres during the growth phase. While such one-dimensional models perform very well on simple ice growth and melt of typical Arctic sea ice, they are not suited to reproduce strong regional melting features as observed in Fram Strait^58^. Hence, we limit model application to the forward tracking of sea ice starting from the Siberian shelf seas (Fig. S2b).

### Sea ice area flux

Ice area flux estimates provided in Fig. 1c are calculated using CERSAT motion estimates together with CERSAT ice concentration information. Fluxes are estimated along a zonal gate positioned at 82.5°N between 60°E and 180°E for the period 1992–2017 (October–April). The ice area flux at the gate is the integral of the product between the meridional (V) and zonal (U) ice drift and ice concentration. A positive (negative) sign refers to northward (southward) transport of sea ice. Transport (flux) rates are given in km².

### Electromagnetic (EM) sea ice thickness measurements

Electromagnetic (EM) ice thickness observations were carried out during summer in northern Fram Strait and the southern part of the Nansen Basin within the framework of the AWI IceBird program. Measurements (shown in Fig. 1e) were obtained in the months of July and August of 2001, 2004, 2010–2012, and 2016–2018 during two cruises of the German ice-breaker RV *Polarstern* and six airborne campaigns with the German DC3-T research aircraft *Polar-5* and *Polar-6*. EM ice thickness measurements utilize the contrast of electrical conductivity between sea water and sea ice to determine the distance of the instrument to the ice-water interface^59^. In 2001 only ground-based EM (GEM) data were obtained using an instrument pulled on a sledge across the ice^60^. In 2004, measurements were made with an airborne EM (AEM) system towed by a helicopter 20 m above the ice surface. Surveys performed after 2010 were conducted with the research aircraft *Polar-5* or *Polar-6* operating from the Danish Station Nord in Nord-East Greenland^20^. The accuracy of the EM measurements is in the order of ±0.1 m over level sea ice^61^. The AEM thickness data enables us to determine the general thermodynamic and dynamic boundary conditions of ice formation^62,63^. The most frequently occurring ice thickness, the mode of the distribution, represents level ice thickness and is the result of winter accretion and summer ablation. We assume the bias that arises from the unknown snow thickness to be negligible, since temperatures above freezing had certainly led to a significantly reduced snow cover or no snow cover at all^56^. For details about data processing and handling we refer to^20,59^.

### Sedimentary budget

Quantification of sediment uptake and transport by sea ice is challenging due to the poorly understood entrainment processes and patchy distribution of matter^12^. A few studies exist that provide values for sediment content in sea ice obtained during expeditions carried out in early to mid 1990s^64,65^. Based on data from various authors^66^ postulates that 10–50% of the total Arctic sea ice is covered by dirty sea ice, and that the average sediment content in Arctic sea ice is ~20–30 mg l^−1^. In the central Arctic and Fram Strait sediment concentrations of up to 40% were found, which emphasizes the significance of sea ice as a long-distance transport agent^67^.

Following^3^, the annual sea ice sediment uptake in the Kara Sea and East Siberian Sea is around 2.4 × 10^6^ t y^−1^ and 1.5 × 10^6^ t y^−1^, respectively (see Fig. S4). The Laptev Sea was found to be the most important source region for sediment-laden ice (10 × 10^6^ t y^−1^), but a more recent study of ^8^ could show that values are much larger and roughly 20–30 × 10^6^ t y^−1^ may be exported from the Laptev Sea towards the central Arctic Ocean. Estimates are based on particle concentrations in ice samples from near-coastal sites and quantifications of new ice formation rates in winter. The authors assume that most sediment-laden ice produced in leads and polynyas reach areas north of the mean multiyear ice edge, and thus will not melt during summer.

Here we use our estimates of FYI survival rate from the forward tracking approach (Tables S1 and S2) together with estimates of sediment uptake by^3,8^ to quantify the amount of sediments released in the marginal shelf seas and central Arctic Ocean before and after 2004 (Fig. S4). Assuming a constant annual flux of matter from the marginal seas, we find that the total amount of sediments released from FYI increased by 24% between 1998–2003 and 2004–2017. This is equivalent to 4.8 × 10^6^ t y^−1^ of mainly fine-grained material with high silt content^12^.

## Supplementary information

Supplementary Info

## Data Availability

Sea ice thickness measurements from the AWI IceBird program are available via the project’s homepage: https://www.awi.de/en/science/climate-sciences/sea-ice-physics/projects/ice-bird.html. Results from the tracking experiments were uploaded to PANGAEA and will be available soon. The gridded thickness fields from the radar altimeters onboard the Envisat and CryoSat-2 satellite platforms are available at the CCI Data Portal^51,52^.
