# Supplementary material for: Arctic warming interrupts the Transpolar Drift and affects long-range transport of sea ice and ice-rafted matter
Source: Sci Rep. 2019 Apr 2;9:5459. doi: 10.1038/s41598-019-41456-y (PMC6445075; doi:10.1038/s41598-019-41456-y)
Supplement: Supplementary file 1 — Supplementary Info [file 41598_2019_41456_MOESM1_ESM.pdf]

Thomas Krumpen[1,*], H. Jakob Belter[1], Antje Boetius[1], Ellen Damm[1], Christian Haas[1], Stefan Hendricks[1], Marcel Nicolaus[1], Eva-Maria Nöthig[1], Stephan Paul[2], Ilka Peeken[1], Robert Ricker[1], Rüdiger Stein[1]

[1]Alfred Wegener Institute, Helmholtz Centre for Polar and Marine Research, Am Handelshafen 12, 27570 Bremerhaven, Germany.

[2]Ludwig Maximilians University, Department of Geography, Luisenstraße 37, 80333 Munich, Germany

Corresponding author: Thomas Krumpen, Thomas.krumpen@awi.de

# Arctic warming interrupts the Transpolar Drift and affects long-range transport of sea ice and ice-rafted matter

## Supplements

### Figures S1 – S4

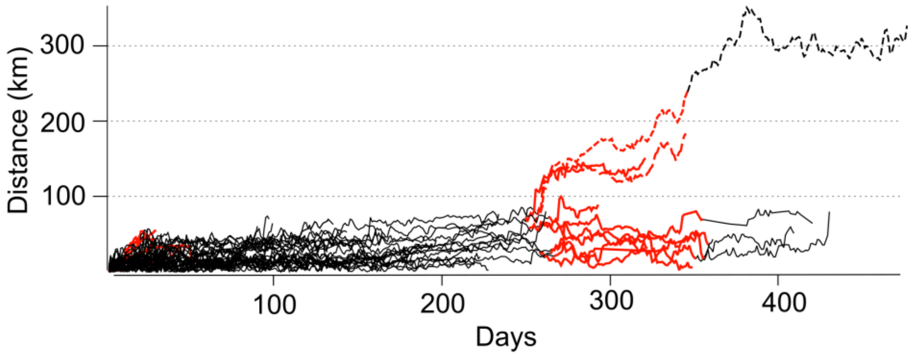


**Fig. S1**: Distance between 57 buoys deployed on sea ice in the Arctic Ocean between 2011 and 2016 and their reconstructed trajectories (virtual buoys). Data from buoys that enter Fram Strait (south of 81°N) are indicated by dashed lines. Sommer months (June – August), when OSISAF or NSIDC data are applied, are marked in red.

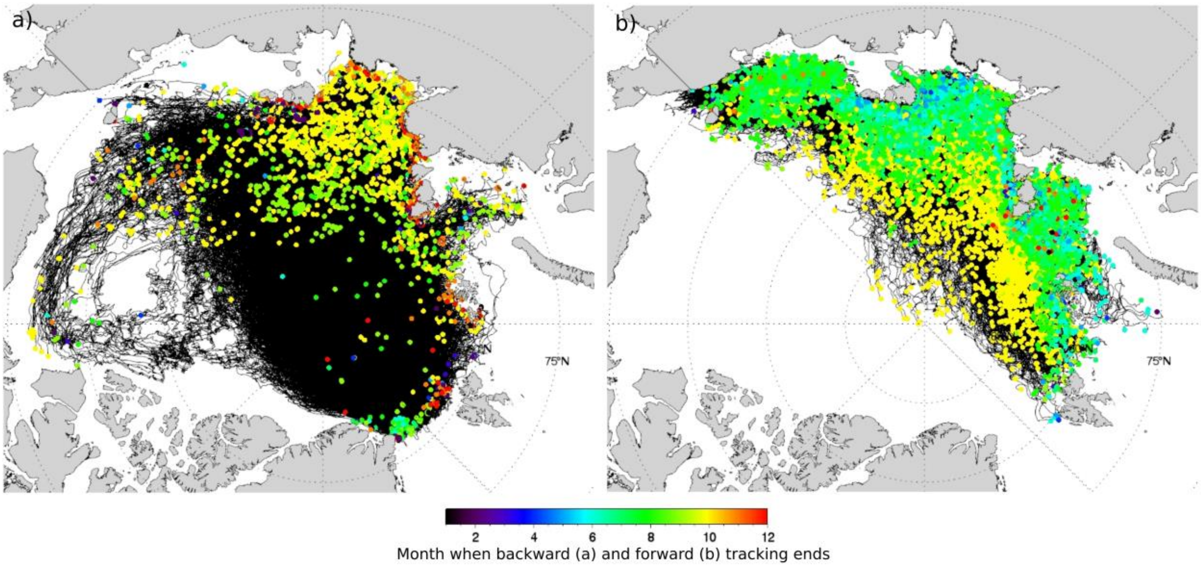


**Fig. S2**: a) Backward trajectories of sea ice (black lines) leaving the Arctic Ocean through Fram Strait between 1998 – 2017 (January – December). Tracking stops when ice reaches land, fast ice or ice concentration drops below 20 %. b) Forward trajectories of sea ice formed in winter (October – April) in shallow waters of the Siberian coast between 1992 – 2017. In forward mode, tracking stops after first summer (freeze-up) or if sea ice concentration is lower than 20 %. In a) and b) the end nodes of the individual tracks are represented by colored circles. The color coding corresponds to the month when tracking stopped.

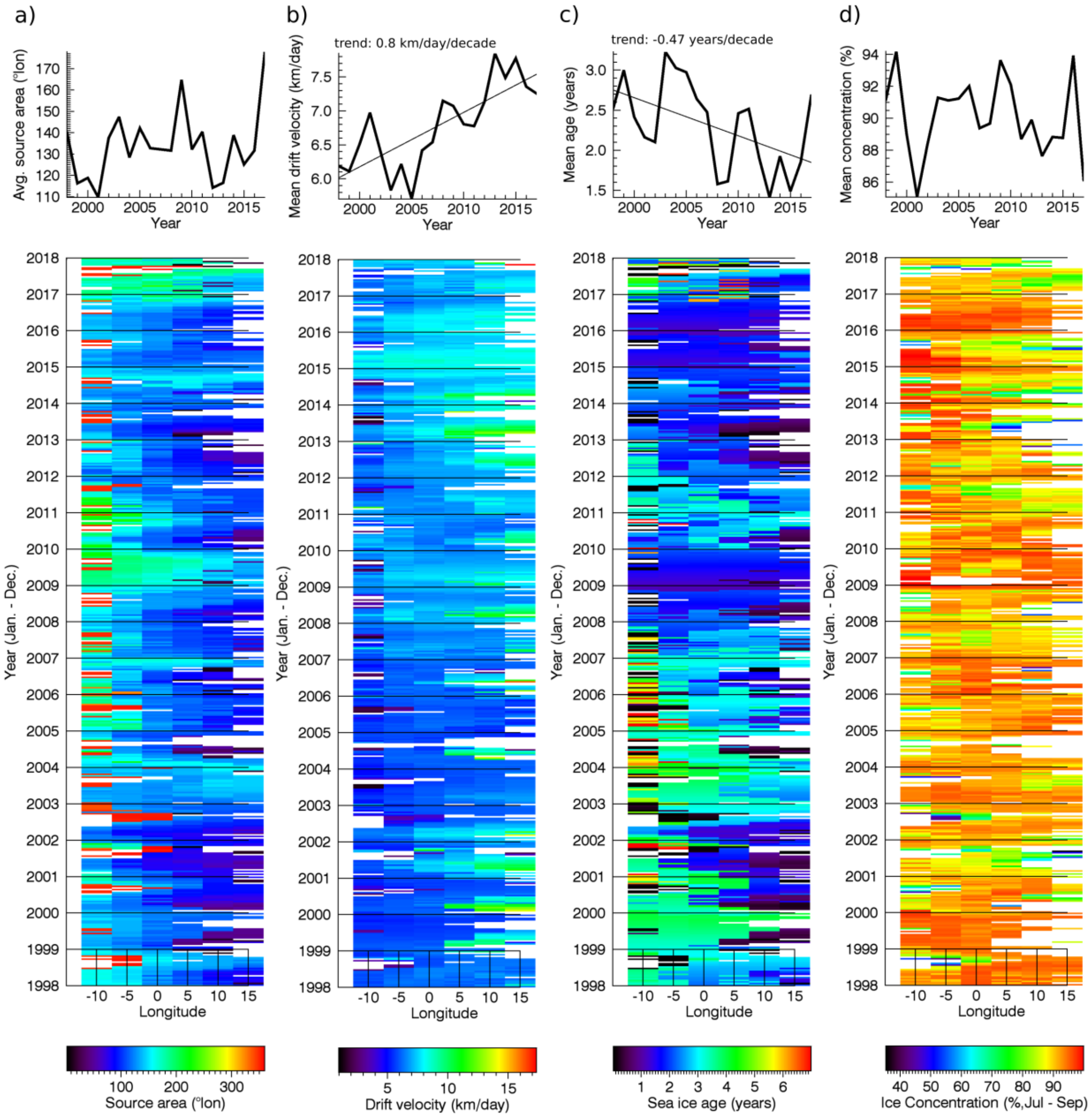


**Fig. S3**: Results from backward-tracking of sea ice starting from 6 locations in Fram Strait (shown in **Fig. 1b**) between 1998 – 2017. Tracking was initiated at a two-week interval. a) shows the source areas of sea ice leaving Fram Strait (compare **Fig. 2e**). The upper panel provides the annually averaged source area, whereas the lower panel shows source areas for individual starting points in Fram Strait (x-axis) and months when tracking was initiated (y-axis) as a Hovmoeller diagram. b) provides averaged along-track drift velocities of sea ice. The age of Fram Strait sea ice is given in c). For b) and c) trends are significant and trendlines are plotted on top. d) shows the mean summer (July – September) ice concentration along trajectories.

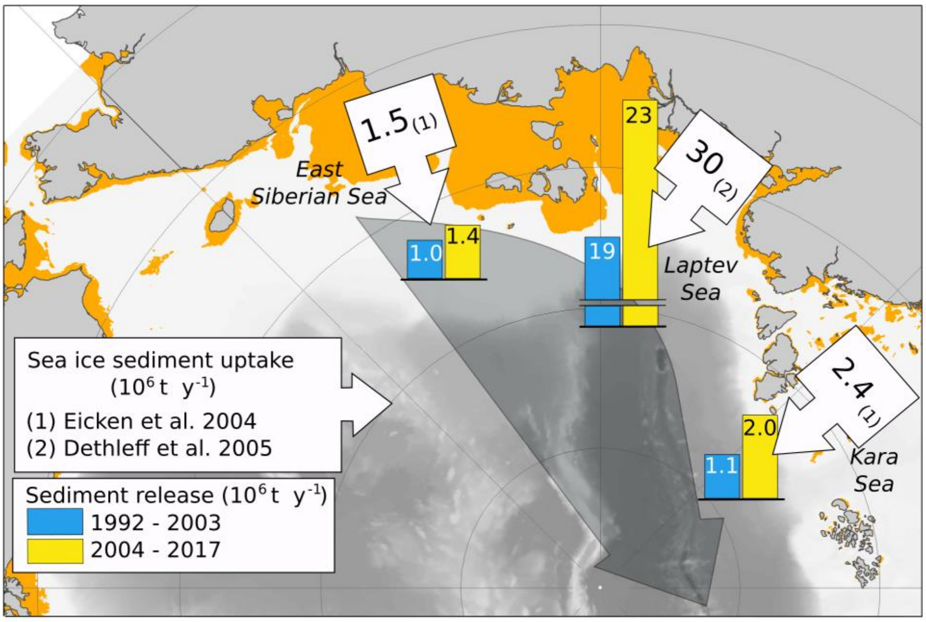


**Fig. S4**: Uptake of sea ice sediments (white boxes) in the marginal seas (based on [1, 2, 3]). Based on the survival rates obtained from the forward-tracking experiment (see **Tab. S2**), we calculated the amount of sediments released by FYI coming from the Laptev Sea, East Siberian Sea and Kara Sea for the two different periods (blue: 1992 - 2003 and yellow: 2004 - 2017).

# Tables S1 – S2

| Location | Site # | Latitude | Longitude | Survival Rate (%) Mean | Max. | Min. | Trend (%/decade) | Significat at 95 % confidence level |
|---|---|---|---|---|---|---|---|---|
| Kara Sea | 1 | 80.9°N | 74.8°E | 29 | 85 | 0 | -31.78 | Significant |
| Kara Sea | 2 | 79.9°N | 79.2°E | 31 | 100 | 0 | -33.43 | Significant |
| Kara Sea | 3 | 78.8°N | 81.9°E | 29 | 100 | 0 | -24.62 | Significant |
| Kara Sea | 4 | 77.7°N | 84.3°E | 22 | 100 | 0 | -21.30 | Significant |
| Kara Sea | 5 | 77.2°N | 88.8°E | 16 | 100 | 0 | -11.54 | Significant |
| Kara Sea | 6 | 78.5°N | 92.0°E | 17 | 92 | 0 | -21.66 | Significant |
| Severnaya Zemlya | 7 | 79.6°N | 89.6°E | 39 | 100 | 0 | -24.79 | Significant |
| Severnaya Zemlya | 8 | 80.7°N | 89.5°E | 54 | 100 | 0 | -33.43 | Significant |
| Severnaya Zemlya | 9 | 81.8°N | 91.3°E | 68 | 100 | 0 | -35.98 | Significant |
| Severnaya Zemlya | 10 | 81.3°N | 98.3°E | 59 | 100 | 0 | -25.15 | Significant |
| Laptev Sea (West) | 11 | 80.2°N | 101.9°E | 41 | 100 | 0 | -10.00 | Not Significant |
| Laptev Sea (West) | 12 | 79.1°N | 105.8°E | 39 | 85 | 0 | -7.46 | Not Significant |
| Laptev Sea (West) | 13 | 78.3°N | 109.5°E | 48 | 85 | 0 | -17.81 | Significant |
| Laptev Sea (West) | 14 | 77.3°N | 113.1°E | 38 | 92 | 8 | -9.11 | Significant |
| Laptev Sea (South) | 15 | 76.1°N | 115.5°E | 28 | 100 | 0 | -5.38 | Not Significant |
| Laptev Sea (South) | 16 | 75.1°N | 118.3°E | 23 | 77 | 0 | -0.77 | Not Significant |
| Laptev Sea (South) | 17 | 74.8°N | 122.7°E | 21 | 77 | 0 | -0.77 | Not Significant |
| Laptev Sea (South) | 18 | 74.2°N | 127.3°E | 11 | 38 | 0 | -0.47 | Not Significant |
| Laptev Sea (South) | 19 | 74.4°N | 132.6°E | 7 | 38 | 0 | 2.37 | Not Significant |
| Laptev Sea (South) | 20 | 74.7°N | 136.4°E | 5 | 38 | 0 | 2.54 | Not Significant |
| Laptev Sea (East) | 21 | 76.0°N | 135.9°E | 32 | 85 | 0 | -1.54 | Not Significant |
| New Siberian Islands | 22 | 76.8°N | 139.9°E | 47 | 92 | 0 | -1.72 | Not Significant |
| New Siberian Islands | 23 | 76.2°N | 143.4°E | 34 | 85 | 0 | 0.18 | Not Significant |
| New Siberian Islands | 24 | 75.9°N | 147.9°E | 33 | 77 | 0 | -8.22 | Not Significant |
| New Siberian Islands | 25 | 75.8°N | 152.6°E | 35 | 92 | 0 | -11.54 | Not Significant |
| East Siberian Sea | 26 | 74.9°N | 156.3°E | 15 | 100 | 0 | -13.14 | Significant |
| East Siberian Sea | 27 | 72.9°N | 157.1°E | 11 | 100 | 0 | -16.45 | Significant |
| East Siberian Sea | 28 | 72.5°N | 161.0°E | 15 | 100 | 0 | -24.97 | Significant |
| East Siberian Sea | 29 | 72.0°N | 164.7°E | 17 | 100 | 0 | -24.38 | Significant |
| East Siberian Sea | 30 | 71.3°N | 168.9°E | 11 | 100 | 0 | -18.05 | Significant |
| East Siberian Sea | 31 | 71.2°N | 172.8°E | 19 | 100 | 0 | -22.01 | Significant |
| East Siberian Sea | 32 | 71.9°N | 176.9°E | 41 | 100 | 0 | -30.00 | Significant |

**Tab. S1**: Summary of the forward tracking experiment of sea ice formed along the Siberian coastline. Mean, minimum and maximum survival rates (%, 1998 - 2017) are given for individual sites (1-32, compare **Fig. 1b**). In addition, trends (% per decade) and outcome of significance tests (95 % confidence level) are provided.

| | | | FYI survival rate | | Mean survival rate (%/period) | | Sediments ($10^6$ t $y^{-1}$) released by FYI | | Sediments ($10^6$ t $y^{-1}$) advected towards Fram Strait | |
|---|---|---|---|---|---|---|---|---|---|---|
| Site # | Shelf Sea | Sediment uptake ($10^6$ t $y^{-1}$) | Trend (%/decade) | Significat at 95 % confidence level | 1992-2003 | 2004-2017 | 1992-2003 | 2004-2017 | 1992-2003 | 2004-2017 |
| 1-9 | East Siberian Sea | 1.5 * | -18.69 | Significant | 35.7 | 9.2 | 1.0 | 1.4 | 0.5 | 0.1 |
| 10-23 | Laptev Sea | 30 ** | -5.4 | Not Significant | 34.9 | 23.6 | 19.4 | 22.9 | 10.5 | 7.1 |
| 24-32 | Kara Sea | 2.4 * | -26.35 | Significant | 53.8 | 15.7 | 1.1 | 2.0 | 1.3 | 0.4 |
| 1-32 | All | 33.9 | -15.07 | Significant | 41.5 | 17.7 | **21.5** | **26.3** | **12.3** | **7.6** |

* Eicken et al. 2004

** Dethleff et al. 2005

**Tab. S2**: The table provides sea ice sediment uptake rates in the marginal seas based on [1, 2, 3], FYI survival rates (trends and averaged values for the two periods), and the amount of sediments released by FYI in the marginal seas and central Arctic Ocean or transported further towards Fram Strait.

[1] Eicken, H. *The role of Arctic sea ice in transporting and cycling terrigenous organic matter. In: R. Stein & R. W. Macdonald (Eds), The organic carbon cycle in the Arctic Ocean*. Springer-Verlag, Heidelberg, (2004).

[2] Dethleff, D. *Journal of Geophysical Research: Oceans* **110**(C7) (2005). C07009.

[3] Stein, R. *Arctic Ocean Sediments: Processes, Proxies, and Paleoenvironment*, volume Volume 2. Elsevier Science, 1 edition, (2008).
